# Public Knowledge, Perceptions and Practices in the High-Risk Lightning Zone of South Africa

**DOI:** 10.3390/ijerph18147448

**Published:** 2021-07-13

**Authors:** Inocent Moyo, Sifiso Xulu

**Affiliations:** 1 Department of Geography and Environmental Studies, University of Zululand, KwaDlangezwa 3886, South Africa; 2 Department of Geography, University of the Free State, Phuthaditjhaba 9869, South Africa; xulus@ufs.ac.za

**Keywords:** lightning, risk perception, community, uMkhanyakude District Municipality

## Abstract

Lightning activity is a hazard for human societies and the environment, and a common feature of South Africa’s climate system, although with great regional variation. The north-eastern section of the country, including the predominantly rural uMkhanyakude District Municipality, is among the most vulnerable regions, where a growing trend of lightning-related deaths and injuries has been observed in recent years. Despite this, and the Disaster Management Act (No. 57 of 2002), which mandates the implementation of hazard and risk assessments at all scales, no detailed research on the community risk perception of lightning incidents has yet been conducted, although such information could help to facilitate mitigation strategies. This explorative study involved a questionnaire survey of 150 community members that aimed to address this gap. Our results suggest that lightning is a real danger and the community had failed to effectively and successfully respond to its various socio-economic implications at the community and individual levels; this demonstrated the vulnerability of the community to the lightning activity in the study area. The contribution of this study is the identification and explanation of how lightning is regarded, understood, interpreted and responded to and how this information provides an opportunity for effective government intervention.

## 1. Introduction

Globally, lightning activity is a natural event that causes numerous wildfires, deaths and injuries to people, and economic damage to infrastructure, communication networks and electrical systems [1,2]. Its destructive character is essentially attributable to massive electrical potential differences and currents that are discharged either as cloud-to-cloud or cloud-to-ground flashes [2,3,4]. As such, research on lightning activity has received renewed interest as global warming is expected to rise, which could intensify convection and thunderstorms [5], leading to more lightning [6]; this is a serious concern for countries at high risk for lightning. Given its complex nature, a better understanding of lightning’s effects on natural and built environments, particularly on lightning-vulnerable societies, require multidisciplinary knowledge and expertise, including climatology, engineering and social sciences [7]. This complexity has enabled researchers to probe and monitor various aspects of lightning repercussions [8], but in this study, we restricted our focus to a community’s risk perceptions that are directly related to lightning activity over a lightning-prone region of South Africa. In other words, we aimed to show that public knowledge about lightning (the actual danger and mitigation possibilities) and people’s views and defenses relating to it are useful and important to the extent of assisting in the implementation of effective disaster mitigation and adaptation strategies. This means that it is not possible to design and implement effective strategies to respond to lightning and lightning strikes without an understanding of community perceptions and responses to it. It is this issue of public knowledge and perception in the study areas that has not received scholarly research; this contribution fills this gap in knowledge about community perceptions and practices and indeed informing disaster response strategies.

South Africa exhibits high rates of lightning-induced deaths (averaging 200 per annum), placing it as the third-highest country in the world after India and the USA [9]. The country’s mortality rate is said to be four times higher than the global average [7], with more deaths recorded in rural settings. In most areas with high lightning-related deaths and injuries, people are commonly affected as they shelter under perilous structures [10]. Various factors that contribute to these casualties include (a) inadequate lightning-safe structures where people stay, work and study; (b) involvement of people in high-risk activities, such as labor-intensive agricultural work; (c) fewer easily available fully enclosed metal-topped vehicles; and (d) a lack of educational programs that emphasise lightning safety [11,12,13]. The same cannot be said about developed countries, with the result that lightning fatality incidents are low while frequently occurring in the developing countries with low adaptive capacities [11,14]. In South Africa, despite rapid urbanisation over the last two decades, most people still inhabit the rural regions or poorly built structures in the urban areas [15]. Thus, information about the frequency, severity and impact of lightning incidents remains a pressing scientific and societal need that holds much promise for the formation of proper protection strategies.

**Figure 1:**
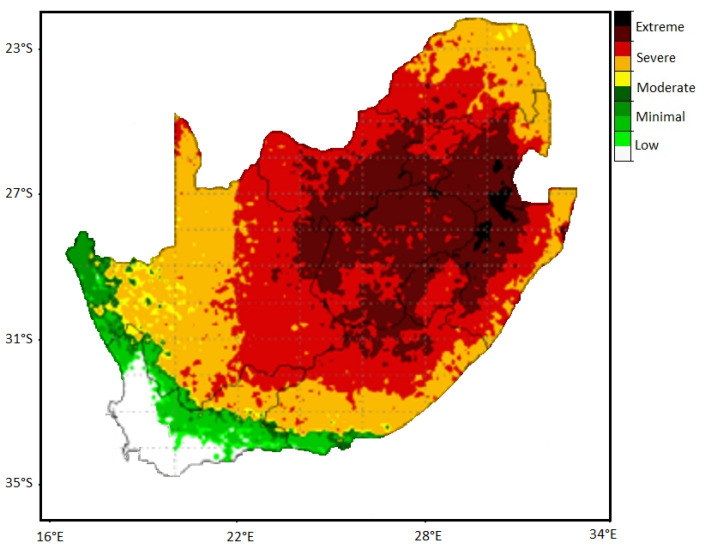
Representation of lightning risk in South Africa computed from high volumes of lightning and lightning with positive polarity data (2006–2010) (modified from Gijben [16], copyright permission has been received).

Our knowledge about the spatial and temporal pattern of lightning events over South Africa improved considerably after the installation of the South African Lightning Detection Network (SALDN) in 2005 by the South African Weather Service [16]. The SALDN is among only three ground-based lightning detection networks in the Southern Hemisphere, with the other two set in Brazil and Australia [17]. The Council for Industrial and Scientific Research (CSIR) had previously produced lightning maps for the country [18], but these appear to have considerably underestimated the frequency of lightning ground density compared to the SALDN, which has displayed far higher results [19]. Consequently, Gill [15] proposed the production of the country’s positive lightning risk map to address the risk associated with lightning; the improved version developed by Gijben [16] is displayed in Figure 1.

As illustrated in Figure 1, the north-eastern section of the country, including the uMkhanyakude District Municipality (UKDM) in the province of KwaZulu-Natal, is among South Africa’s most vulnerable regions, with severe to extreme lightning risk (flash densities of more than 15/km^2^) [16]. As expected, this is the area where many lightning fatalities and injuries are often reported in the media. Actually, no other region of South Africa has as high a lightning risk as in the north-eastern parts of the country.

The reality of lightning-induced fatalities and injuries has awakened community leaders to call for the installation of lightning rods to protect vulnerable community members in the rural areas in UKDM (https://www.algoafm.co.za/domestic/ifp-calls-for-lightning-rods-to-be-installed-in-rural-areas; accessed on 20 December 2020). Despite the assertion by the Disaster Management Act (No. 57 of 2002) that hazard and risk assessments must be carried out at local to national scales [20], as well as the high number of reported deaths and injuries caused by lightning in this area, there is, as yet, no detailed research that surveyed community’s risk perception toward lightning activity.

The need to understand how communities in disaster-prone areas perceive risk has been highly emphasised, as this could aid the formation of mitigation strategies [21,22]. By definition, risk perception entails people’s judgements and evaluations of threats to which they are exposed and influences their acceptance of risk and their conduct before, during and after a disaster [23]. This is tightly associated with social demographics, risk knowledge and disaster experience [24]. Furthermore, it is determined by the public’s trust in and the accuracy of early warning systems [25]. In their empirical study in Canada, for example, Silver and Conrad [26] established that if the community perceives an early warning as inaccurate or inflated, they are more reluctant to take preventative measures than if they consider the information to be trustworthy. Taken together, inaccurate perceptions of disastrous weather events can greatly amplify the vulnerability of communities exposed to them [26]. While several studies on public risk perceptions of lightning incidents (e.g., [11,12,14,27,28]) were conducted in different parts of the world, in this study, we aimed to gain a general understanding of the community risk perceptions and attitudes (descriptions of and responses to actual lightning experiences) towards lightning incidents in the uMkhanyakude District Municipality. We also examined the strategies that are used to cope with lightning.

## 2. Materials and Methods

### 2.1. Study Area

**Figure 2:**
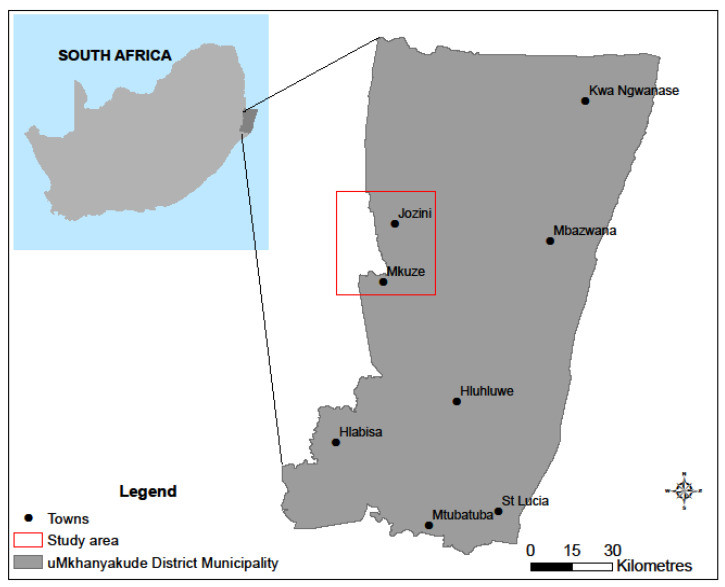
Location of the study area within the uMkhanyakude District Municipality in South Africa.

This study was undertaken in the Jozini and Mkhuze areas within uMkhanyakude District Municipality in the northern KwaZulu-Natal province of South Africa (Figure 2). As is the case with most rural communities in KwaZulu-Natal, traditional authority politically governs the Mkhuze and Jozini area under the Jozini Local Municipality.

### 2.2. Explorative Approach and Agenda

In this study, we adopted an exploratory research approach to ascertain the community’s perception towards the risk related to lightning hazards and, in the process, uncover their attitude on this subject. Exploratory research involves examining social reality on which there is limited research and/or the issue has not been fully delineated. In other words, “researchers explore when they have little or no scientific knowledge about the group, process, activity, or situation they want to examine but nevertheless have reason to believe it contains elements worth discovering. To explore effectively a given phenomenon, they must approach it with two special orientations: flexibility in looking for data and open-mindedness about where to find them” [29]. Based on this, the aim of exploratory research is to survey social reality and, in so doing, provide a better understanding that could be the foundation for detailed and conclusive research [30,31]. Informed by the principle that exploratory research is flexible, in this study, a qualitative approach was followed. The qualitative approach is naturalistic and interpretative [32] and it employs inductive logic regarding how people (in this case, the community members in the study area) understand and interpret their lived experiences [32,33]. This is why questionnaires were used to collect data from community members in this study. These questionnaires allowed for the quantification of certain elements (thus further demonstrating the flexibility of exploratory research) while offering an opportunity for the overall qualitative investigation of the study. This is an issue that is explained further below.

### 2.3. The Questionnaire and the Sample

Both Jozini and Mkhuze are located in Jozini Local Municipality, which in 2016, had a population of approximately 198,215 [34]. Since this is essentially a qualitative study that follows non-probability sampling theory, the determination of the sample size does not follow a formula or steps but instead involved carefully selected informal sampling frames or guidelines [35]. These guidelines include sample sizes reported in journals and other reports [35]. In phenomenological research, sample sizes of between 6–10 people are generally considered to be adequate [32,35]. With this in mind, a total sample of 150 community members was selected using convenience sampling. This was considered adequate in terms of meeting the objectives of exploratory research, as explained in the preceding section. Following Jackson’s [36] assertion that the community’s capacity to understand disasters is intimately linked to disaster experience and knowledge, we considered only community members who had resided in the area for at least five years. This was to allow for deeper insights, as these community members were expected to have rich historical experience on the subject of this study. The actual process of data collection entailed a snowball sampling technique, where respondents were asked to suggest the target households. Concerning the data collection instrument, the authors surveyed the literature on community perception of natural hazards and risks, with specific reference to lightning activity, after which, a structured questionnaire was formulated. The final version of the questionnaire contained 20 questions that were segmented into four sections: demographic attributes, perceptions, strategies for responding to lightning and attitudes and expectations of the local authorities. Of the total, 8 questions were closed-ended and 12 were open-ended. The questions were systematically worded and organised to encourage respondents to reflect on their perceived risks associated with lightning activity. The actual data collection therefore involved oral face-to-face conversations with the 150 community members (questionnaire interviews) between January and February 2021.

### 2.4. Statistical and Thematic Analysis

The data analysis started with the quantitative (closed-ended) data, which were coded into Statistical Package for Social Sciences (SPSS) and computed using descriptive statistics. This was then followed by the qualitative (open-ended) responses, which were evaluated with a thematic analysis [31]. This involved an inductive approach in terms building “patterns, categories and themes from the bottom up, by organizing the data into increasingly more abstract units of information” [31]. This was achieved by reading through the completed questionnaire (open-ended part of the questionnaire) data and then coding it, which allowed for the identification and description of major themes on lightning in the study area. The final steps in the analysis involved an explanation and connection of the themes [32] to matters of public or community risk perceptions on lightning in UKDM.

## 3. Results and Discussion

The analysis of the results was primarily based on the perceptions and attitudes of 150 respondents who participated in the study, an amount that is consistent with several previous works conducted on community perceptions of natural hazards (e.g., Silver and Conrad [26]). The results are structured into four sub-sections: demographic attributes of respondents, their knowledge of lightning activity and related coping strategies, as well as their risk perceptions, after which, the strengths and limitations of the study are presented.

### 3.1. Demographic Characteristics of Respondents

A larger proportion (60%) of respondents in this study comprised females, and the majority ranged between 19 and 40 years of age (Table 1). This could be accounted for by noting that, in general, men tended to migrate to the towns and cities in search of employment, leaving behind women, children and the elderly. However, some studies actually indicate that the male-dominated migration patterns are slowly changing as more women are also engaged in migration as much as men [37]. Here, the respondents were dominated by young adults from 19 years to adults reaching 50 years.

**Figure 3:**
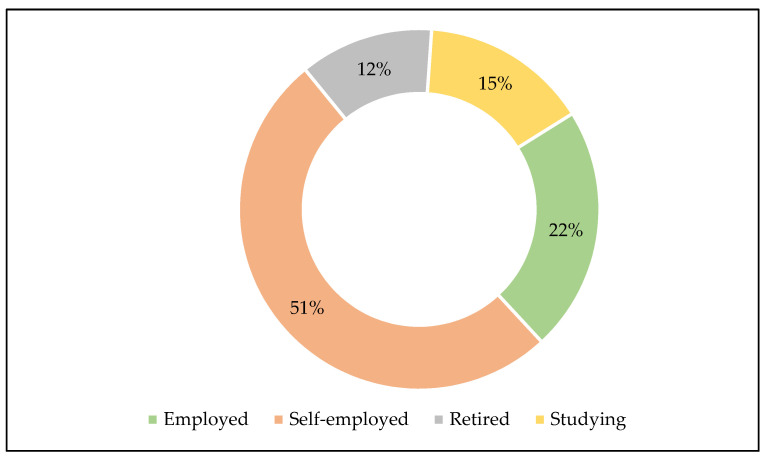
Livelihood activities.

In the studied area, the respondents were involved in diverse livelihood activities (Figure 3), slightly half (51%) of which were in self-employment (including farming), followed by those who were formally employed (22%), studying (15%) and pensioners (12%). Although farming may be considered as either formal employment (e.g., farm workers) or self-employment (e.g., small farming enterprises that provide both food and income to the owner), in this study, the respondents were not involved in farming in a formal capacity, such as farm workers. Concerning educational attainment, the majority of the respondents had a secondary school level of education.

In addition to this, Table 2 shows that over 88% of the respondents had lived in the area for more than 10 years. Those who had lived in the study for 6 to 10 years constituted 7%, and 5% of the respondents had lived in the area for not more than 5 years

Taken together, the age, educational attainment, duration of stay and the livelihood activities of the respondents suggested that they had relatively grounded experiences and perceptions of lightning, including its fatalities in the study area. For example, the fact that the majority of the respondents had lived in the study area for more than a decade highlights that their views and experiences captured the actual reality on the ground since they had actual encounters with lightning and its effects for as long as they had been alive. Some of these encounters with lightning were actually related to the livelihood activities of these community members, such as self-employment and farming.

Likewise, the high literacy rates also point to the fact that the respondents interacted with the researchers with a deeper understanding of the issues under discussion. Based on this, the views and experiences that are discussed in the following parts can be considered to be a true reflection of the community’s perception of the risk linked to the lightning hazard and their coping strategies.

### 3.2. Respondents’ Knowledge of Lightning

From the questionnaire, the majority (96%) of respondents stated that they had a clear knowledge of lightning activity, while few (4%) seemed to have inconclusive knowledge of this sinister phenomenon. Follow-up questions on the kind of knowledge of lightning that the respondents had yielded two main views: 51% indicated that they were only aware of the prevention of lightning damage but did not understand its causes and 45% of the respondents indicated that they knew the causes and methods for preventing lightning damage. Regarding the causes of lightning, explanations by the respondents ranged from accurate scientific accounts to the realm of superstition. Concerning the former, the respondents identified the causes of lightning to include, among others, natural causes, climate changes and high temperatures. It should be noted that concerning the latter, the community members asserted that witchcraft was prevalent in the study area and, therefore, it was a misnomer to classify cultural explanations of lightning as superstition (interviews with community members (no. 12–no. 57), Jozini, January 2021). The community members who held these views were convinced that some members had magical powers that allowed them to cause lightning to strike their enemies. For example, one community member explained that once those who possessed magical powers were unhappy with a prosperous neighbour or community member, they simply engineered a lightning strike. According to this explanation, the severity of lightning engineered in this manner depended on the depth of conflict, in which minor conflicts were accompanied by light lightning strikes to warn and/or scare off the enemy. However, if the conflict was deep and entrenched, the lightning strikes were always fatal in terms of killing both people and livestock and the strikes destroyed property (interview with a community member (no. 77), Mkuze, January 2021). These views on the cultural origins and/or causes of lightning confirm other comparable studies, such as one from Trengove and Jandrell [28], who established that in some African societies, there was a very strong belief that lightning can be controlled by witches/wizards to kill people and their livestock.

#### 3.2.1. Respondents’ Risk Perception before, during and after Lightning Incidents

**Figure 4:**
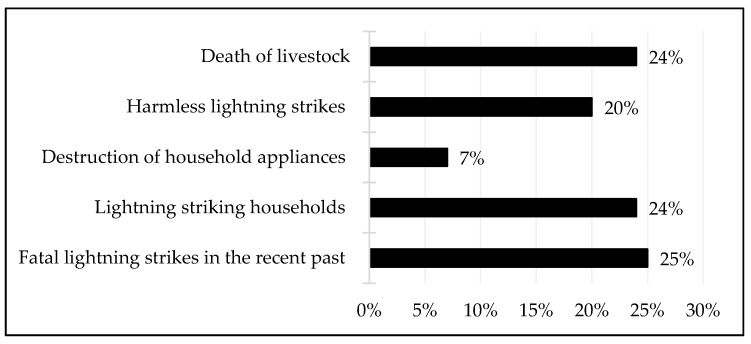
Experience of lightning strikes and their consequences.

In addition to exploring the respondents’ knowledge of lightning, it was considered important to understand the experiences of community members regarding lightning activity and its consequences. In this respect, the results show that some people have died, houses or household appliances were struck and destroyed by lightning and livestock were killed by lightning, among other consequences (Figure 4). According to the interview results, fatal lightning strikes affected all age groups and genders, but it was mostly the adult males who were frequent victims, whose susceptibility was linked to the livelihood activities in which they engaged. For instance, almost all men who had been struck by lightning were working on their farms or herding and/or looking for livestock in the veld (interview with a community member (no. 104), Mkhuze, January 2021). This shows that it is more the activities that community members engaged in and less their gender that exposed them to lightning strikes. A related issue is that some of the young people who had also been struck by lightning were those engaged in outdoor activities, such as walking to or from school or playing in lightning-prone areas (interviews with community members (no. 58–no. 117), Mkhuze, January 2021). Indeed, this confirmed the observations by Elsom and Webb [38] that gender is not a fundamental reason for the risk of lightning; rather, it is the type of activity being undertaken when it occurs that is more important. Moreover, research evidence shows that men tend to participate in activities that are considered at high risk of being struck by lightning than women. In terms of age, the highest fatality rates were amongst the 20–29-year-olds, many of whom were associated with outdoor activities [38].

The fact that it became clear during interview conversations that it was mostly men and young adults (school-going age) who were most at risk of being struck by lightning due to the activities they engaged in led the researchers to further probe these categories of people so as to understand why they continued to engage in activities that exposed them to lightning. Men stated that farming and domesticating livestock was how they earned livelihoods and there was simply no way they could stop doing this (interview with community members (no. 1–no. 11), Jozini, January 2021). In this regard, one community member asserted that even if they had knowledge and/or early warning systems about the prevalence of lightning, they could not stop farming or domesticating their livestock because doing so would lead to food shortages and death, i.e., another disaster (interview with a community member (no. 9), Jozini, January 2021). Therefore, these community members were trapped or entangled in an intractable dilemma: either to stay indoors and go hungry or go out and farm and tend to livestock and be struck by lightning. Likewise, the school-going children could not stop attending school because of lightning, whether it was actual or potential. They had to go to school and unfortunately play in lightning-prone areas. In these situations, we see that the school-going children, just like the men discussed in the preceding section, were faced with a dilemma. They could not stop going to school even if rain accompanied by lightning was threatening. While at school, they could not stop themselves from playing. It was the nature of children to play and this was duly recognised by school authorities, who allocated breaks for lunch that the school-going children utilised for eating and also playing outdoors (interviews with community members (no. 118–no. 150), Mkhuze, January 2021). The point we are making here is that it was traveling to school and engaging in outdoor activities that caused school-going children to lose their lives to lightning and yet they could not stop engaging in these activities. In any event, safety at home was not always fully guaranteed because some of the homesteads were also struck by lightning (interview with a community member (no. 2), Jozini, January 2021).

Those respondents who reported that they lost family members as a result of lightning strikes described such a loss as traumatic and deeply painful. One respondent explained that they lost their father to a lightning strike and, since that time, “life has never been the same because there is no one to take care of the family and we generally struggle more than what used to be the case when our father was alive. In short, life is now difficult” (interview with a community member (no. 150), Mkhuze, January 2021). Such lightning-related deaths were a yearly occurrence and this unsettled many people because they lived in fear that they could also die whenever lightning struck. Thus, even though most of the respondents had knowledge about lightning, as well as some preventive strategies, and some believed that these prevention strategies were effective, this did not remove the fear of fatal lightning strikes in the study area. As a result, 93% of the respondents indicated that they did not feel safe; this was particularly the case whenever there was heavy rainfall accompanied by thunderstorms. This fear was fueled by two main issues. First, the frequency and intensity of lightning strikes in the study area, and second, the role of witchcraft’s utilisation of lightning to settle personal and other feuds. Concerning the first issue, the respondents shared that it increased the chances of any one of them being struck by lightning. In other words, the more incidents of lightning strikes, the higher the chance that a person could be fatally struck. On the second matter, the respondents stated that this was complicated by the fact that one was never certain if they had enemies who had magical powers that allowed them to use lightning to solve conflicts. Consequently, whenever there were violent storms accompanied by lightning, “one starts to think that perhaps they wronged another member of the community who had decided to take action by launching a lightning strike” (interview with a community member (no. 127), Mkhuze, January 2021). This fear led to 94% of the respondents stating that they felt unsafe. To this, the loss of property and, in particular, livestock can be added. A respondent who reportedly lost a significant number of cattle to a lightning strike in 2020 stated that such a loss negatively affected the livelihood of the family. This was because they had lost potential income from the sale of livestock, and without the livestock, there was no such income (interview with a community member (no. 127), Mkhuze, January 2021). For example, livestock was sold to buy food, pay school fees and meet other family costs.

In the recent event referred to in the preceding part in which livestock was killed as a result of lightning, this meant that all these costs were not met, which made life generally harder and led to poverty. In all this, it can be seen that the lightning strikes in the study area had socio-economic ramifications. However, it is important to stress that although similar impacts of lightning could be experienced in other parts of the world, including Europe and North America, the extent of the impact in the South African region under consideration is without comparison, with obvious consequences in terms of safety. This was precisely because of the low level of preparedness by the community or poor and inadequate infrastructure to respond to a lightning disaster. Indeed, this is consistent with observations by other studies, such as [10,11,12,13], where factors that contribute to high numbers of lightning-related fatalities are linked to, among others, poor lightning infrastructure. Furthermore, the socio-economic ramifications of lightning discussed in the preceding part had implications on community health. For example, poverty meant that people could not access food, resulting in malnutrition. Similarly, the death of livestock, such as cattle, resulted in a loss of milk and meat, which have necessary nutrients for healthy living. This meant that people survived on foods that had limited nutrients and this had potential impacts on their health. In addition to this, the fact that people lived in perpetual fear that they could die at any time, especially during the rainy season, had implications on health. Although this study did not engage in an exploration of the mental health implications of lightning strikes on the community, interview conversations revealed that people were stressed or apprehensive, particularly during the rainy season. Likewise, whenever clouds gathered in the sky, people became afraid that they could be victims. This stress or apprehension is a matter of health, bearing in mind the definition by the World Health Organisation (WHO) [39] that health “is a state of complete physical, mental and social well-being and not merely the absence of disease or infirmity” [39]. Of course, the connection between lightning strikes and health is a matter that needs more in-depth investigation for conclusive results to be attained.

#### 3.2.2. Respondents’ Coping Strategies

**Figure 5:**
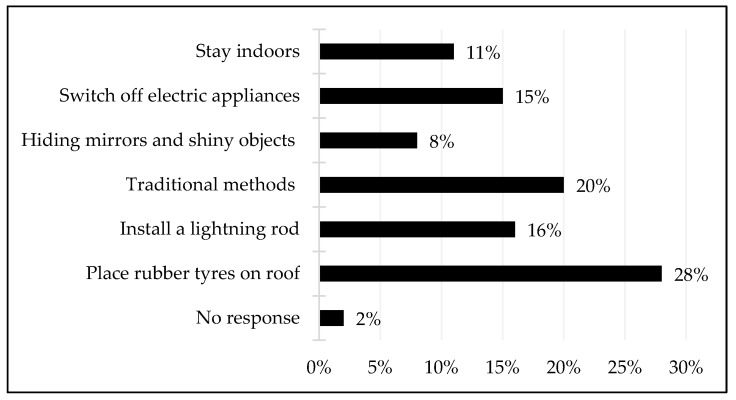
Lightning prevention strategies.

Beyond accounting for causes of lightning, which were divided between scientific and cultural explanations, a related issue was that of how the community members responded to the lightning strikes or how they protected themselves from lightning. Figure 5 shows that the strategies that were employed by the community members included scientific and traditional and/or religious approaches. This can be demonstrated by the fact that 28% of the participants stated that they placed rubber tyres on their rooftops since they believed this method prevented their houses from being struck by lightning. This is why one community member stated that “I place rubber tyres on my rooftop because I believe that rubber has the ability to deflect lightning” (interview with a community member (no. 140), Mkhuze, January 2021). Based on scientific information/knowledge, other participants indicated that they had installed lightning rods in their houses to prevent lightning fatalities. These respondents shared that the lightning rods were lightning conductors, which protected people, livestock and property from being killed or destroyed by lightning strikes. Further, some respondents explained that they used traditional methods to protect themselves from lightning; this involved the sprinkling of traditional medicine, which was believed to deflect lightning strikes away from their houses and other properties. Traditional methods also included the planting of certain trees around the homestead, which were believed to have supernatural powers in terms of preventing the lightning from striking. This is why one participant asserted that “I have planted trees (*umuthi*) around the yard to prevent lightning damage” (interview with a community member (no. 133), Mkhuze, January 2021).

It should also be added that other community members believed in the power of Christian prayer because “nothing beats the protection of prayer, so when there is lightning, we pray as a family” (interview with a community member (no. 145), Mkhuze, January 2021). Other strategies that were utilised by the community members so as to protect themselves from lightning strikes included hiding shiny objects and covering mirrors during a rainstorm. This was also thought to be an effective strategy for preventing lightning strikes because mirrors and shiny objects are believed to attract lightning. Some participants stated that they switched off all electric appliances and gadgets during lightning since electric appliances are thought to attract lightning. In addition, other participants stated that they stayed indoors to avoid being struck by lightning; this was aptly captured in a discussion with one respondent who stated that “when it is raining, I minimise movement and stay indoors to be safe” (interview with a community member (no. 141), Mkhuze, January 2021).

The preceding discussion on the lightning prevention strategies also raised the related question of the extent to which these were effective. In this regard, all the respondents had absolute faith in their chosen lightning prevention strategies, including the traditional approaches. On the question of why there were frequent and fatal lightning strikes if the various lightning strategies were efficacious, one respondent was adamant that “it could be worse if we did not utilise the various traditional strategies, but the fact of the matter is that in cases where lightning did strike, it could be because people failed to follow instructions in full. In cases where instructions were followed in full, there was guaranteed protection. In short, I can say the key lies with whether or not people followed instructions of how to protect themselves against lightning” (interview with community member (no. 133), Mkhuze, January 2021). In addition to this, it was posited that in cases where lightning strikes were caused by very powerful witches/wizards, they were difficult to control. This was precisely because it was believed that the person engineering the lightning deliberately orchestrated it to be extremely powerful so that any preventive measures, including both traditional and scientific responses, were futile; otherwise, the objective of the lightning strike would not be achieved (interview with a community member (no. 133), Mkhuze, January 2021).

### 3.3. Respondents’ Expectations of Authorities

**Figure 6:**
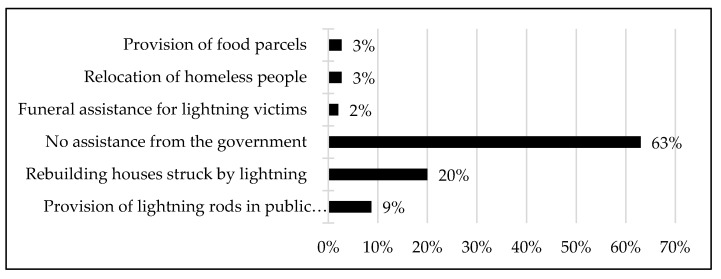
Interaction of the community with the local government.

The discussion on the response by individual community members in terms of coping strategies to lightning strikes suggests that there was a need for government intervention. This raised the question of the role of and interaction between the local government and the community. A crucial aspect of this community–local government interaction was communication. In this respect, the respondents indicated that there were various methods that they used to communicate with the local government. These included communication through community meetings, traditional authority and the ward councillor. However, 22% of the respondents stated that it was difficult to access or communicate with the local government and the same respondents also asserted that they were not familiar with the lines of communication between them and the local government. Although the majority of the respondents (78%) indicated that there was communication with the local government, as indicated in the preceding, the fact that some community members felt left out, even if they were few, is worrisome. This is because we posit that in areas that are frequently affected by disasters, such as this study area, the lines of communication between all community members and the government must be clear and known. For those who stated that they communicated with the local government, they indicated that this communication included, among others, reporting lightning incidents to the municipality. This raised the question of how the local government responded to such reports by the community. The results of this study show that the municipality assisted the community by installing lightning rods in public spaces and institutions, such as schools. In addition, it was reported that the local government helped by rebuilding houses that were destroyed by lightning and also relocated homeless people after lightning-related destructions. Further, in some cases, the local government also assisted in covering the funeral costs of victims in order for them to have a decent burial (Figure 6).

It was also reported that the government was involved in education programmes in which they taught the community in the study area about lightning and the personal- and community-level preventative measures that needed to be implemented so as to avoid lightning fatalities. Despite these government efforts, 63% of the respondents were of the view that the government intervention programmes were not effective. The ineffectiveness was based on the belief that, among others, it took too long for the government to respond to lightning strikes and, in most cases, it took fatal lightning strikes for the government to intervene; as such, one respondent added that “the government only acts when someone died due to a lightning incident” (interview with a community member (no. 50), Jozini, January 2021). It was because of this perceived ineffectiveness of government intervention regarding lightning strikes and their fatalities in the study area that the respondents highlighted several expectations of the government. These included the need for the government to expand its educational campaigns to all members of the community. This was considered important so that all people had the correct information on lightning, namely, its causes and effects, as well as possible preventive measures. The community also suggested that the government should widen its installation of lightning rods beyond public places and institutions. It should now be clear that the expectations from the community members included programmes that were already being done by the government but it was the contention of the community that these programmes did not reach all parts of the study area. For example, the community members had problems with the fact that lightning rods were installed in public places and institutions and yet people spent most of their time in their homes. Thus, while it was a good thing to install lightning rods in schools, for example, it was not effective because people did not stay in schools and clinics, but instead in their homes, and that is where the preventive strategies needed to be implemented and/intensified. The full meaning is that it was the expectation from the community members in the study area that both public places and spaces, such as schools, and private ones, such as houses/homesteads, needed to have lightning rods installed.

## 4. Limitations and Outlook

Our study is not without caveats. First, we considered only community members that had stayed in the area for over five years. This could have excluded some community members who recently relocated to the study area and yet could have had useful information. Second, the data collection was conducted during the COVID-19 lockdown in South Africa and some of the respondents were not always willing to respond to the questions. In fact, the researchers were prevented from entering some of the homesteads/houses. As a result, some interviews were conducted across the fence/wall because of the fear of the transmission of the coronavirus. Despite this, all the data that was required to respond to the aim of the study was collected. Lastly, future research might consider a detailed demographic and time–space analysis of the lightning-related fatalities to explain the most vulnerable cohort and the associated spatiotemporal pattern. Related to this is the need for future research to assess the impact of lightning strikes and fatalities on the health of communities in the study area.

## 5. Conclusions

In the final analysis, it needs to be emphasised that lightning activity in the study area was perceived as a dangerous phenomenon that was caused by natural forces, as well as human activity in the form of witchcraft. In other words, well before lightning struck, the community was aware that their area of residence was exposed to lightning and related risks. This is why before lightning strikes occurred, community members fortified their houses/homesteads by sprinkling traditional medicine or planted trees that were perceived to intercept lightning. Some of the community members did not believe in the cultural explanations (witchcraft) of lightning and this is why they installed lightning rods on their houses, as well as implemented other scientifically informed strategies of responding to lightning. Both those who used traditional and scientific responses to lightning were not always successful, as lightning struck frequently, claiming lives, destroying property or both. This was further complicated by the fact that some of the activities, such as farming and domesticating livestock, which mostly males were mostly involved in and exposed them to lightning, were mandatory. There were no alternatives to farming and/or domesticating livestock. People had to either farm and domesticate livestock or stay at home and die of hunger; the latter was not an option. This led people to engage in livelihood activities that exposed them to lightning.

In this lies the vulnerability of the community to the lightning activity in the study area: the community had failed to contain lightning strikes and its various socio-economic implications at the community and individual levels. An example of this is the death of livestock due to lightning, which led to the loss of income and hence poverty. To this can be added the widespread fear whenever there were rainstorms because people were never certain if they would come out of such rainstorms alive. This is why the respondents in this study were of the view that the government had abandoned them to the extent that the government concentrated on installing lightning conductors in public places, such as schools and clinics, and not on the homes of people.

Differently stated, the community expected the government to engage in a comprehensive household-level installation of lightning conductors so that every person was at least prepared for the lightning strikes. According to the respondents, this needed to be accompanied by a comprehensive education programme (in the form of, for example, an easily usable and brochure in the local language) of every community member on the causes and consequences of lightning and the possible coping strategies. This means that the community expected more active and comprehensive and/or widespread government involvement in preparing for and responding to lightning activity in the study area than is currently the case. Consequently, by exploring public risk perceptions on lightning activity in UKDM, which is a high-risk lightning zone in South Africa, the contribution of this study involved identifying and explaining the gaps around how lightning is regarded, understood and/or interpreted and responded to, and how these gaps provide an opportunity for comprehensive and effective government intervention. For example, based on the fact that half of the respondents did not subscribe to scientific explanations of the causes and response to lightning, this is the gap that must be addressed by the government through relevant education programmes. Likewise, the issue of the provision of lightning conductors in public places provides an opportunity for the government to consider providing lightning conductors to all households/homesteads or at least partner with communities in this regard. This is the context within which we posit that hazard and risk assessments must be implemented at all scales, in line with the proclamation by the Disaster Management Act (No. 57 of 2002). A study like this one that explores community risk perception of lightning is useful and/or needed so as to both inform and facilitate effective and comprehensive mitigation strategies in a setting like that of UKDM, which is endemically gripped by disastrous lightning activity.

## Figures and Tables

**Table 1:** Respondents’ gender and age distribution.

Age Categories (Years)							
		≤18	19–30	31–40	41–50	≥51	Total
Gender	Female	11 (7)	24 (16)	23 (15)	20 (13)	12 (8)	90 (60)
	Male	7 (5)	19 (13)	14 (10)	13 (9)	7 (5)	60 (40)
	Total	18 (12)	43 (29)	37 (25)	33 (22)	19 (12)	150 (100)

Figures in parentheses are percentages and those out of parentheses are frequencies.

**Table 2:** Respondents’ duration of stay in the area.

Duration of Stay	Frequency	Percentage
≤5 years	7	5
6–10 years	11	7
≥11 years	132	88

## Data Availability

To protect the confidentiality of research participants, data are not publicly available.
